# A gate-free monolayer WSe_2_ pn diode

**DOI:** 10.1038/s41467-018-05326-x

**Published:** 2018-08-07

**Authors:** Jhih-Wei Chen, Shun-Tsung Lo, Sheng-Chin Ho, Sheng-Shong Wong, Thi-Hai-Yen Vu, Xin-Quan Zhang, Yi-De Liu, Yu-You Chiou, Yu-Xun Chen, Jan-Chi Yang, Yi-Chun Chen, Ying-Hao Chu, Yi-Hsien Lee, Chung-Jen Chung, Tse-Ming Chen, Chia-Hao Chen, Chung-Lin Wu

**Affiliations:** 1 Department of Physics, National Cheng Kung University, Tainan, 70101 Taiwan; 2 Department of Materials Science and Engineering, National Tsing Hua University, Hsinchu, 30013 Taiwan; 3 Department of Electrophysics, National Chiao Tung University, Hsinchu, 30010 Taiwan; 4 Department of Materials Science and Engineering, National Chiao Tung University, Hsinchu, 30010 Taiwan; 5 Center for Micro/Nano Science and Technology, National Cheng Kung University, Tainan, 70101 Taiwan; 6 National Synchrotron Radiation Research Center (NSRRC), Hsinchu, 30076 Taiwan

## Abstract

Interest in bringing p- and n-type monolayer semiconducting transition metal dichalcogenides (TMD) into contact to form rectifying pn diode has thrived since it is crucial to control the electrical properties in two-dimensional (2D) electronic and optoelectronic devices. Usually this involves vertically stacking different TMDs with pn heterojunction or, laterally manipulating carrier density by gate biasing. Here, by utilizing a locally reversed ferroelectric polarization, we laterally manipulate the carrier density and created a WSe_2_ pn homojunction on the supporting ferroelectric BiFeO_3_ substrate. This non-volatile WSe_2_ pn homojunction is demonstrated with optical and scanning probe methods and scanning photoelectron micro-spectroscopy. A homo-interface is a direct manifestation of our WSe_2_ pn diode, which can be quantitatively understood as a clear rectifying behavior. The non-volatile confinement of carriers and associated gate-free pn homojunction can be an addition to the 2D electron–photon toolbox and pave the way to develop laterally 2D electronics and photonics.

## Abstract (web summary)

Bringing together p- and n-type monolayers of semiconducting transition metal dichalcogenides results in the formation of atomically thin pn junctions. Here, the authors laterally manipulate carrier density to create a WSe_2_ pn homojunction on a supporting ferroelectric BiFeO_3_ substrate.

## Introduction

Devices that require low power consumption, materials that have a monolayer structure with quantum confinement, electrons and holes that convey information with high mobility—these are just some breakthroughs that might be realized following the development of two-dimensional (2D) materials that are efficient, scalable, and easily engineered to achieve diverse functionality. Since the discovery of various 2D materials almost a decade ago, the recent boost of interest in semiconducting layered transition metal dichalcogenides (TMD) originates from their exotic characteristics in the monolayer limit, such as giant spin-valley coupling^1,2^, optical control of valley polarization and coherence^3,4^, an indirect-to-direct bandgap transition^5–7^ and tightly bound excitonic states^8–10^. Despite these fantastic discoveries, the present challenge is to promise a TMD pn diode, which is a fundamental building block of modern devices, that manifests all their numerous advantages (including monolayer structure, homo-interface, and device functionality) while also easily overcoming the scaling limit of current complementary metal-oxide semiconductor (CMOS) technology or achieving atomically thin optoelectronics. The methods used to achieve p- and n-doping in TMD are mainly inducing charge transfer to TMD, such as gate-bias tuning^11–13^, interacting with atoms/molecules^14–17^, molecular adsorption on a surface^18,19^, and plasmonic hot-electron doping^20,21^. The method most commonly used to dope TMD is to set a gate voltage through the metal gating, which provides a direct way to realize the strong charge-density tuning, but metal gates would result in an inhomogeneous charge distribution and unavoidable degradation of the emission of light at the TMD surface. TMD diodes have been constructed by stacking vertically, such as an ionic liquid-gated bulk MoS_2_ device^22^, a TMD/III–V semiconductor^23^ and a TMD/doped-silicon^24^. TMD heterojunctions vertically involving other materials, however, apparently lack many appealing exotic properties of a lateral monolayer.

**Fig. 1:**
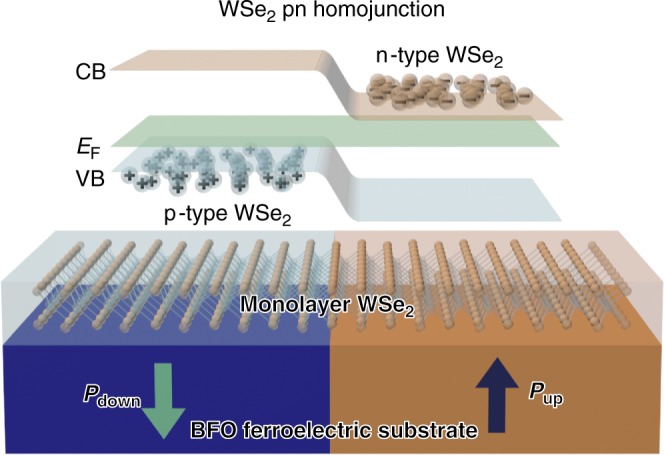
Schematic band diagram of a WSe_2_ pn homojunction derived from a ferroelectric-pattern-assisted BFO layer. Both polarization states (*P*_down_ and *P*_up_) on a ferroelectric BFO layer can directly affect the carrier type of monolayer TMD with either p-type or n-type semiconducting behavior

Because of the ultra-thin nature of a TMD, a necessary substrate provided by a functional material can offer a strategy for lateral modulation of the TMD band structure without problems caused by the doping defects and the mismatches between the dopants and 2D lattice atoms. Ferroelectric (FE) materials possess a spontaneous electrical polarization that can be macroscopically and locally inverted with an external stimulus, and are considered to be prospective substrates to support TMD to achieve a pn homojunction, as the accumulation or depletion of an inevitably charged mobile carrier occurs in the TMD to screen the polarization field of the FE substrate. Here, using detailed spatially resolved spectroscopies, we have demonstrated the respective WSe_2_ electron-filling (n-type) and electron-emptying (p-type) regions configured and modified with the FE domains of a BiFeO_3_ (BFO) substrate, and thus define a monolayer WSe_2_ pn homojunction (sketched in Fig. 1). The current flowing through a WSe_2_ pn diode is non-volatilely rectified with a source-drain voltage (*V*_SD_) without assistance of gate biasing. This diode shows a strong current-rectifying behavior in electrical transport properties, which confirms the results revealed in the homojunction band structure. This work provides a non-volatile control of TMD doping and a promising way to produce a pn homojunction as a future building block of 2D device applications.

## Results

### Scanning probe microscopy and μ-PL characterization

**Fig. 2:**
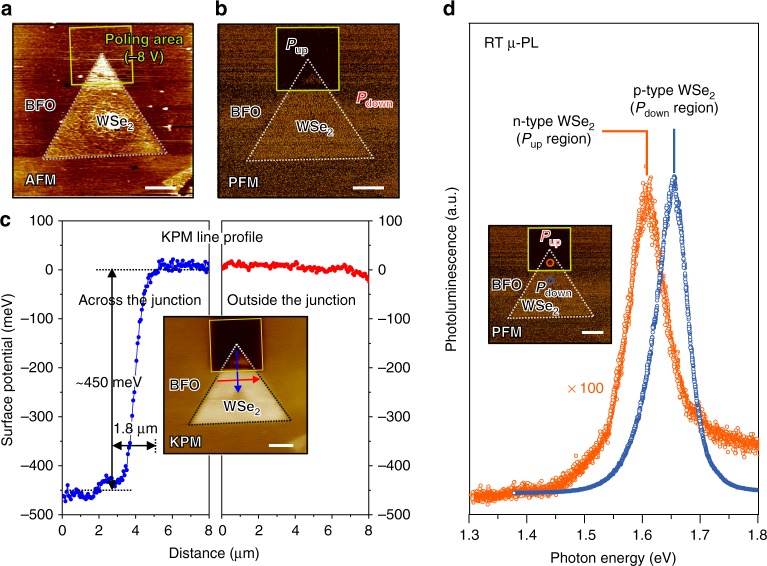
The scanning probe microscope images and μ-PL spectra of the WSe_2_ pn homojunction. WSe_2_ on *P*_up_ and *P*_down_ regions from measurements with an AFM and PL. **a**, **b** AFM and PFM taken of a WSe_2_ sheet on the BFO ferroelectric layer as grown. The solid line shows poled regions with bias −8 V at an AFM tip (yellow lines). A PFM image taken of a WSe_2_ sheet on a BFO layer with *P*_up_ and *P*_down_ regions, which shows the out-of-plane ferroelectric polarization in BFO to have phase difference 180°. **c** KPM image and line profile image taken across and outside the *P*_up_ and *P*_down_ homojunction. **d** PL of a WSe_2_ sheet taken from the *P*_up_ and *P*_down_ homojunction. The scale bars in the figures are 5 μm

The crystalline ferroelectric BFO layers were grown with pulsed-laser deposition (PLD) on (001) SrTiO_3_ (STO) substrates with a conductive SrRuO_3_ (SRO) layer (Methods section). Using chemical-vapor deposition (CVD) and a wet transfer method, we affixed monolayer crystalline WSe_2_ sheets firmly to the BFO surface, forming a van der Waals (vdW) interface that played a key role in the formation of a WSe_2_ homojunction. The scanning-probe characterizations and images of a representative WSe_2_ on ferroelectrically patterned BFO are displayed in Fig. 2. As in the case of the scanning line profile, the thickness of the WSe_2_ was a monolayer (~1.5 nm) measured by tapping-mode atomic force microscope (AFM) and confirmed by photoluminescence (PL). The monolayer thickness of WSe_2_ measured here was larger than the mechanically exfoliated monolayer WSe_2_ (*d*_WSe2 _~ 0.7 nm)^19^ and the CVD-grown monolayer WSe_2_ (*d*_WSe2_ ~ 1.1 nm)^25,26^ since the water molecules were easily trapped on the hydrophilic BiFeO_3_ surface to increase the distance between WSe_2_ and BiFeO_3_ substrate^27,28^ and thus increase the thickness measured by AFM. The ferroelectric properties of the BFO layer were verified through characterization with polarization versus voltage (*P*–*V*) and a piezo-force microscope (PFM) in Supplementary Figure 1 and Fig. 2b, respectively. The *P*–*V* loops show that the BFO films used in this work having naturally downward polarization (*P*_down_) undergo sharp FE switching during poling, with a remnant polarization ≈60 μC cm^−2^ (Supplementary Figure 1)^29^. To demonstrate the ferroelectric control of WSe_2_-doped charges, we created a ferroelectric domain pattern on reversing the polarization through scanning with a metal probe (probe voltage set to −8 V) to obtain an area of upward polarization (*P*_up_) that is partially covered with WSe_2_; the overlap area is about 7 μm^2^. In the PFM image shown in Fig. 2b, two distinct *P*_up_ and *P*_down_ regions are revealed under a WSe_2_ sheet with opposite out-of-plane phases; the shape of the WSe_2_ sheet is consistent with that measured from an AFM image, implying that the WSe_2_ sheet is not structurally damaged during the reversal of polarization.

To reveal the substrate-induced charge traps in WSe_2_, which can significantly alter the surface potential and work function of WSe_2_, measurements with a Kelvin probe microscope (KPM) showed that the surface potential of WSe_2_ decreases about 450 meV because of the induced charge screening in WSe_2_ within the *P*_up_ area, compared within a *P*_down_ area as shown in Fig. 2c. This potential difference of WSe_2_ (~0.45 eV) created with ferroelectric *P*_up_ and *P*_down_ regions of BFO is significantly larger than with an epitaxial growth of monolayer WSe_2_-MoS_2_ lateral junction^30^, which shows that the ferroelectricity of the supporting substrate has the ability to affect the electrical properties of TMD material efficiently and strongly. Moreover, the monolayer WSe_2_ has a direct band gap that offers a large quantum yield of radiative exciton recombination, leading to efficient PL emission. These photo-excited electrons and holes can form either excitons (e–h pairs) or electron-/hole-bounded trions (e–e–h/e–h–h) even near 300 K, and, accordingly, can be used to monitor the carrier type (doping type) and density in a TMD having direct bandgap emission^31^. The spatially resolved PL spectra recorded from the same area (circle in Fig. 2d) of the monolayer WSe_2_ sheet at two opposite polarization states is depicted in Fig. 2d. It is clearly visible that the pronounced PL emission induced a red shift in position and a significant attenuation of intensity from the natural *P*_down_ state (~1.65 eV) to the reversed *P*_up_ state (~1.60 eV). This is consistent with the fact that the emission switched to decreased energy and quantum yield from a hole-bounded trion recombination to an electron-bounded trion recombination in the initially p-type WSe_2_ and then becoming electron-accumulated WSe_2_ under the *P*_up_ state of the BFO substrate, which is in agreement with previous reports on the PL characteristics of monolayer TMD under electrostatic gating^32^. Moreover, we recorded PL spectra in a series at the same spot on the sheet under another FE switching cycle, which confirmed that the attenuation of the PL emission is not from destruction due to biased tip scanning during FE poling, as shown in Supplementary Figure 2.

### The SPEM/S measurements

**Fig. 3:**
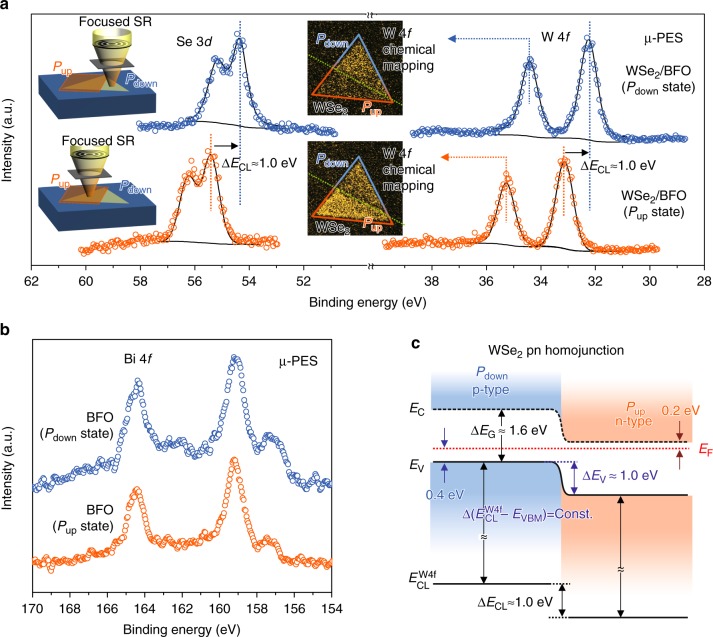
SPEM images and μ-PES measurements on the WSe_2_ pn homojunction. Se 3*d*, W 4*f*, and Bi 4*f* core-level photoelectron spectra measured with SPEM in *P*_up_ and *P*_down_ regions of a WSe_2_/BFO homojunction. **a** Core-level spectra of Se 3*d* and W 4*f* recorded from a *P*_down_ (blue) and a *P*_up_ (orange) region. SPEM images of W 4*f* taken in 34.6 eV and 35.4 eV, which correspond to a *P*_down_ and a *P*_up_ region, respectively. **b** Core-level spectra of Bi 4*f* emitted from the BFO substrate. **c** The band structure deduced from **a** reveals the pn junction for WSe_2_ in *P*_down_ and *P*_up_ regions near 300 K

To directly visualize the ferroelectric tuning in the electronic structure of WSe_2_ on a BFO, we used a scanning photoelectron microscope and spectroscopy (SPEM/S, in National Synchrotron Radiation Research Center (NSRRC), Hsinchu, Taiwan) to provide the required spatial resolution (~sub-μm) for chemical mapping and the energy resolution (± 50 meV) for localized photoelectron spectroscopy (μ-PES) with a beam of synchrotron radiation (SR) focused on the *P*_up_ and *P*_down_ regions larger than the size of the focused SR beam. Figure 3a, b shows Se 3*d*, W 4*f*, and Bi 4*f* core-level photoelectron spectra, which correspond to the SPEM images taken from a WSe_2_ sheet at the *P*_up_ and *P*_down_ regions, respectively. The binding energy (BE) of the core-level electron in Se 3*d* and W 4*f* of the *P*_up_ region was significantly greater than in *P*_down_, but was not observed in Bi 4*f*. For the band structure of the *P*_down_ region sketched in Fig. 3c, the entire Fermi-level energy is located 0.4 eV above the valence band, which displays a p-type semiconductor behavior. An as-grown *P*_down_ BFO layer hence preserved the p-type behavior, because p-type doped BFO thin film has a work function value near that of WSe_2_^33,34^. The detailed band structures deduction of WSe_2_ at *P*_up_ and *P*_down_ are shown in Supplementary Figures 3 and 4. Explicitly, with ferroelectric polarization switching, we observed that Se 3*d* and W 4*f* have a BE shift ~1.0 eV in the *P*_up_ region. This energy shift is observed also in a SPEM image, which reveals a contrast reversal in the W 4*f* images. The energy difference of the core level corresponds to the Fermi-level energy shift in the band gap, which turned the p-type into n-type WSe_2_, as shown in Fig. 3c. The tuning of the Fermi level (*E*_F_) within the gap that is significantly larger than the previously reported number for Nb-doped MoSe_2_ and NO_*x*_-doped WSe_2_^14,16^ implies that it is possible to tune the *E*_F_ position near the TMD band edge with heavily doping according to this approach. Here, this ferroelectricity-assisted band structure engineering results a WSe_2_ pn homojunction at room temperature.

To determine the ferroelectricity-induced manipulation of WSe_2_ carrier density, the PES spectra of WSe_2_ under *P*_up_ and *P*_down_ BFO states are required to reveal the respective *E*_F_ energies relative to their valence (*E*_V_) and conduction (*E*_C_) band edges. Under a parabolic approximation for the band dispersion near the bottom of conduction band (CB) and the top of valence band (VB) modeled in the effective mass of mobile carrier with Fermi–Dirac statistics, the 2D electron density (*σ*_n_) in WSe_2_ is given by *σ*_n_ = (*g*_2D_*k*_B_*T*) ln{1 + exp[(*E*_F_–*E*_C_)/*k*_B_*T*]} and the 2D hole density (*σ*_p_) in WSe_2_ is *σ*_p_ = (*g*_2D_*k*_B_*T*) ln{1 + exp[−(*E*_F_−*E*_V_)/*k*_B_*T*]}, in which *g*_2D_ is the electron/hole density of state for WSe_2_. As shown in Fig. 3c, the *E*_F_–*E*_V_ of WSe_2_ is about 0.4 eV in the *P*_down_ state and *E*_C_*–E*_F_ of WSe_2_ is about 0.2 eV in the *P*_up_ state, which are determined by the spectra shown in the Supplementary Note 3. As a result, the carrier densities in the p-doped and n-doped WSe_2_ regions are estimated of *σ*_p_ ~ 8.63 × 10^9^ cm^−2^ and *σ*_n_ ~ 1.48 × 10^13^ cm^−2^, respectively, and the detailed calculation is provided in the Supplementary Note 6. Comparing the huge electron density tuning range (~10^10^ cm^−2^) based on the intrinsic carrier density (*σ*_i_ ~ 1.26 × 10^3^ cm^−2^) of WSe_2_, the mobile hole accumulation in WSe_2_ is slightly inhibited (~10^7^ cm^−2^) when the polarization in BFO layer is naturally *P*_down_, which is in agreement with the results of the defect charge screening of naturally polarization field (*P*_down_) in BFO proposed in previous results^27^. Due to the large polarization field provided by a ferroelectric BFO substrate, the large improvement of the mobile electron density (~10^13^ cm^−2^) in TMD system observed in this study agrees satisfactorily with the order of surface bound charges of the BFO layer (~10^14^ cm^−2^) and is higher than other TMD junction systems that was reported for elemental doping MoSe_2_ (*σ*_n_ ~ 10^11^ cm^−2^) and lateral heterojunction WSe_2_/MoS_2_ systems (*σ*_n_ ~ 10^10^ cm^−2^)^14,30^. Moreover, the tunability of mobile charge density in 2D TMD system can be probably achieved in precisely manipulating the polarization field in supporting polycrystalline ferroelectric layer through setting different poling voltage, for example, the polarization in polycrystalline BFO can be changed from 5 to 70 μC cm^−2^ thus would modulate the charge density as large as one order of magnitude^35,36^.

### Direct verification of pn homojunction

**Fig. 4:**
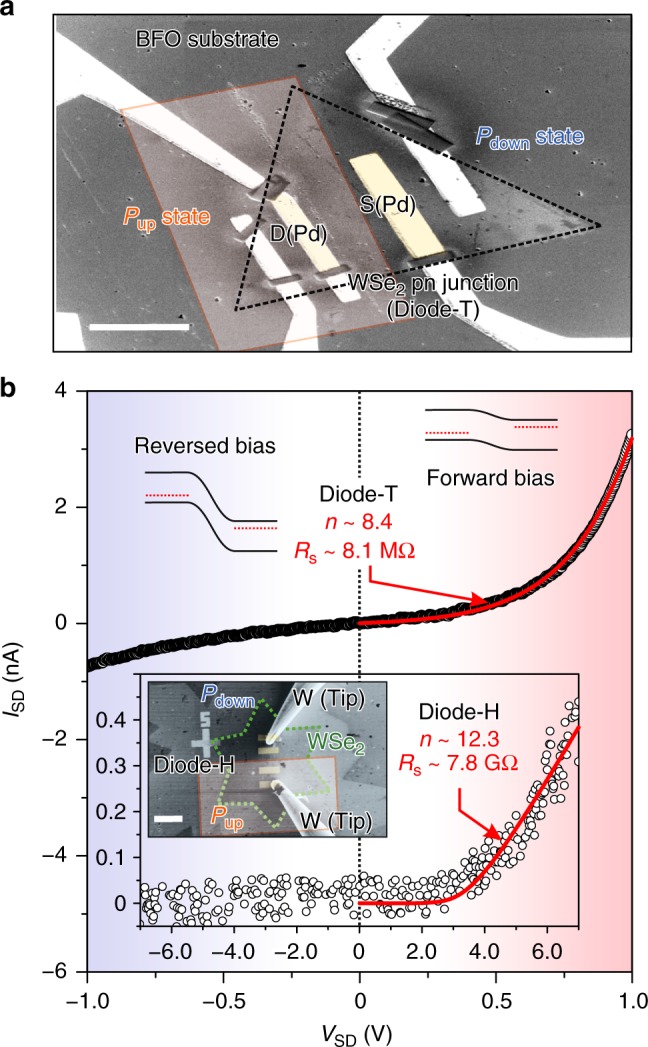
The SEM images of the devices for electrical transport measurements and their corresponding *I*–*V* curves. Measurements of electrical transport of a pn homojunction in WSe_2_/BFO devices. **a** SEM image for a WSe_2_ homojunction (Diode-T) from a top view of the junction. **b** Current measured as a function of voltage for the pn WSe_2_ junctions on BFO (~30 nm, Diode-T) and thicker BFO (~60 nm, Diode-H) layers. The fits of the Shockley equation with extended series resistance at the forward-bias region give the ideality factor *n* and series resistance *R*_s_ of WSe_2_ diodes on thin (~30 nm, Diode-T) and thick (~60 nm, Diode-H) BFO layers. Both scale bars in the SEM images are 10 μm

The spatially resolved spectral measurements provided clear evidence and insight into the ferroelectric control of WSe_2_ carrier densities on a BFO substrate; a question arises whether one can reveal the rectification of a WSe_2_ pn homojunction on reversed BFO domains. The fabrication of the WSe_2_ pn diode (Diode-T) made use of the CVD growth of crystals of WSe_2_, with a monolayered and triangle sheet (area ~100 μm^2^) transferred onto a 30-nm-thick BFO substrate and then employed with the same ferroelectric switching in the AFM setup. Figure 4a presents a scanning electron microscope (SEM) image of the device used to measure the electrical transport measurements. In this way, the WSe_2_ sheet became ferroelectrically doped into p-type (right half) and n-type (left half) conducting regimes to form a WSe_2_ pn homojunction. All transport measurements were performed near 300 K and in vacuum, wherein the voltage bias *V*_SD_ is applied and the direct current *I*_SD_ is measured between source (S) and drain (D) contacts. Here, we choose to use Pd metal to form low-resistance ohmic contacts with WSe_2_ because of its high work function^19^. It is worth noting that the Pd electrode is also found to conduct current into the BFO substrate when patterned upon it. Two conducting tungsten tips controlled by the nanomanipulator installed in SEM are utilized to conduct the electrical measurements. To prevent inevitable BFO substrate effect, the Pd metal electrodes are being cut off connections with BFO substrate using focused-ion-beam etching, which turning into isolated pad on the source and drain points (see Fig. 4a, b inset).

Figure 4b shows current measurements for the WSe_2_ pn homojunction. Under reversed bias, the diffusion potential barrier height between the p-type and n-type sides becomes too high to flow a significant current through the junction and shows rectification behavior characteristic of a classic diode. Such diode *I*_SD_–*V*_SD_ characteristics are typically modeled by the Shockley diode equation with extended series resistance *R*_s_^37,38^,

1 $$I_{\rm{SD}} = \frac{{nV_{\mathrm{T}}}}{{R_{\mathrm{s}}}}W\left[ {\frac{{I_0R_{\mathrm{s}}}}{{nV_{\mathrm{T}}}}{\mathrm{exp}}\left( {\frac{{V_{\mathrm{SD}} + I_0R_{\mathrm{s}}}}{{nV_{\mathrm{T}}}}} \right)} \right] - I_0,$$

where *I*_0_ is the reverse-bias saturation current and *R*_s_ is the series resistance, while *nV*_T_ is the thermal energy at room temperature with the ideality factor *n* (*n* ≥ 1) of the diode. *W* is the Lambert *W* function. At the forward-bias voltages, the *I*_SD_–*V*_SD_ curve of our WSe_2_ pn diode with a homo-interface is well modeled by the diode equation with *n* ~ 8.4, series resistance *R*_s _~ 8.1 MΩ. This ideality factor *n* is quite low compared with what have been observed for the CVD-grown TMD diodes, normally *n* *>* 10^39,40^, however, it is still higher than the *n* value obtained in certain TMD diodes made by exfoliated natural crystal with high-quality^12,41,42^. Furthermore, no obvious reverse-saturation current is observed, indicating that current transmission through the whole WSe_2_/BFO device is not limiting in the diode and suggesting that the BFO layer acts not as a good insulating layer to prevent current leakage as shown in Supplementary Figure 5.

To explore issues of current rectifying associated with the supporting BFO layer quality and to better understand the nature of current transmission through Pd metal contacts and WSe_2_, we made another WSe_2_ diode (Diode-H) consisting of a transferred CVD-grown WSe_2_ monolayer flake having hexagram shape and large area (~700 μm^2^) on a thicker BFO layer (thickness ~ 60 nm) with opposite polarization states (shown in inset of Fig. 4b). Clearly, the current rectification can be reproducibly observed as shown in the *I*_SD_–*V*_SD_ characteristics. The low saturated current (~10^–15^ A, below the 1 pA noise level of the measurement) is extracted from diode equation fitting and can be observed at high reversed bias (0 ~ −5 V), confirming that the thick BFO layer indeed inhibits current leakage. The higher *n* (~12.3) value and larger series resistance *R*_s_ (~7.8 GΩ) based on the fitting result with the turn-on point in the *I*_SD_–*V*_SD_ plots showing at large forward bias is attributed to a high series resistance consistently associated with the poor conductive quality of a hexagram shaped WSe_2_ layer^43–46^. Moreover, on this large WSe_2_ diode, it is easy to make both Pd electrodes on WSe_2_ in *P*_down_ states (pp junction) and in *P*_up_ states (nn junction). Since Pd is a high work function metal^47^, which means its Fermi-level will align valence band edge of WSe_2_ for efficient hole injection, therefore, the pp junction shows a nearly ohmic *I*_SD_–*V*_SD_ relation at low *V*_SD_. The *I*_SD_–*V*_SD_ relation in the nn junction shows non-linear due to the Schottky barrier formed at the n-type WSe_2_/Pd interface, in which shown in Supplementary Figure 6. Apparently, minimizing the series resistance and defect densities including using asymmetric metal contacts on exfoliated crystalline WSe_2_ should significantly improve the performance of this ferroelectricity-assisted WSe_2_ pn diode.

## Discussion

To summarize, we designed and implemented a ferroelectrically controlled WSe_2_ diode using a lateral pn homojunction without a biased electrode gate. Employing a monolayer WSe_2_/BFO structure, we demonstrated non-volatile control of the band structure, surface potential, and light emission of monolayer WSe_2_ with opposite polarization domains on a BFO substrate. With the quantitative analysis and modeling of current rectifying, as device quality improves, our gate-free 2D diode promises a comparable capability of current rectification with that of a conventional bias-gating-type 2D device. This evidence emphasizes the possibility of non-volatile control and provides an alternative functionality in ferroelectric doping of 2D materials, thus opening a wide vista of TMD-based quantum electronics and photonics.

## Methods

### Crystal growth

A tungsten diselenide (WSe_2_) sample was prepared with CVD. On evaporation of WO_3_ at a high temperature, the WO_3_ powders were filled in a home-made quartz reactor with a transfer tube of tunable length to enable a stable flow of WO_3−*x*_ vapor that served as a reactant evaporated from a high-temperature zone to react with Se on a substrate surface. In the low-temperature zone, an individual WSe_2_ monolayer was synthesized in a temperature range 650–750 °C. Monolayer WSe_2_ typically formed a triangular shape in a monolayer thickness to transfer onto the BFO ferroelectric layer.

An epitaxial thin film (30 nm and 60 nm) of BFO was fabricated on the SRO-buffered STO (001) single-crystalline substrate via pulsed-laser deposition (KrF excimer laser, *λ* = 248 nm); the laser beam was focused on a BiFeO_*x*_ ceramic target with energy density ~2.5 J cm^−2^ and repetition rate 10 Hz. The samples were deposited at substrate temperature 700 °C under oxygen at pressure 100 mTorr. The samples were cooled to near 300 K under oxygen at pressure 1 atm after deposition of the thin film. The ferroelectric epitaxial BFO/SRO/STO(001) film possessed out-of-plane downward polarization; the domain structures as grown were confirmed with a PFM.

### Details of SPEM/S measurement

The localized SR-PES technique provides a powerful method to obtain direct information about a band structure with varied polarization; soft X-rays (photon energy 400 eV) were used at the SPEM end station located at beamline 09 A1 of Taiwan Light Source in NSRRC. The soft X-ray beam focused by the Fresnel zone plate and the order-sorting aperture at the focal plane was about 100–200 nm in diameter. All measurements were undertaken near 300 K. The energy resolution was estimated to be better than 100 meV. Based on the SPEM images, the focused beam was movable to a specific location to record high-resolution PES of a microscopic area.

### Data availability

The data that support the findings of this study are available from the corresponding authors upon reasonable request.

## Electronic supplementary material

Supplementary Information

Peer Review File
