# Supplementary material for: A gate-free monolayer WSe2 pn diode
Source: Nat Commun. 2018 Aug 7;9:3143. doi: 10.1038/s41467-018-05326-x (PMC6081376; doi:10.1038/s41467-018-05326-x)
Supplement: Supplementary file 1 — Supplementary Information [file 41467_2018_5326_MOESM1_ESM.docx]

Supplementary Information

for

“A Gate-free Monolayer WSe_2_ *pn* Diode”

Chen *et al.*

**Supplementary Note 1: P-V hysteresis loops with varied measurement frequency of a BFO (001) film**

The macroscopic P-V hysteresis loop with different measuring frequency on BFO (001) films. From Supplementary Figure 1, we can obtain the saturation polarization on BFO film in value of *P_s_*~ 60 μC cm^-2^.

**Supplementary Figure 1**: Macroscopic electric P-V hysteresis loops with varied measurement frequency of a BFO (001) film**^[1]^**.

**Supplementary Note 2: Photoluminescence of WSe_2_ on BFO**

To confirm that the intensity attenuation did not originate from destruction by the tip on scanning with an AFM, we investigated the *P*_up_ and *P*_down_ regions upon BFO layer. We scanned the naturally downward polarization area (probe voltage set to +8 V) and another upward (probe voltage set to -8 V) with the AFM tip. In Supplementary Figure 2, the PL measurement reveals decreased photon-energy emission and intensity attenuation in the *P*_up_ region, compared with the *P*_down_ region. These results confirm that there is no destruction in the poled region of either morphology or piezoelectricity.

**
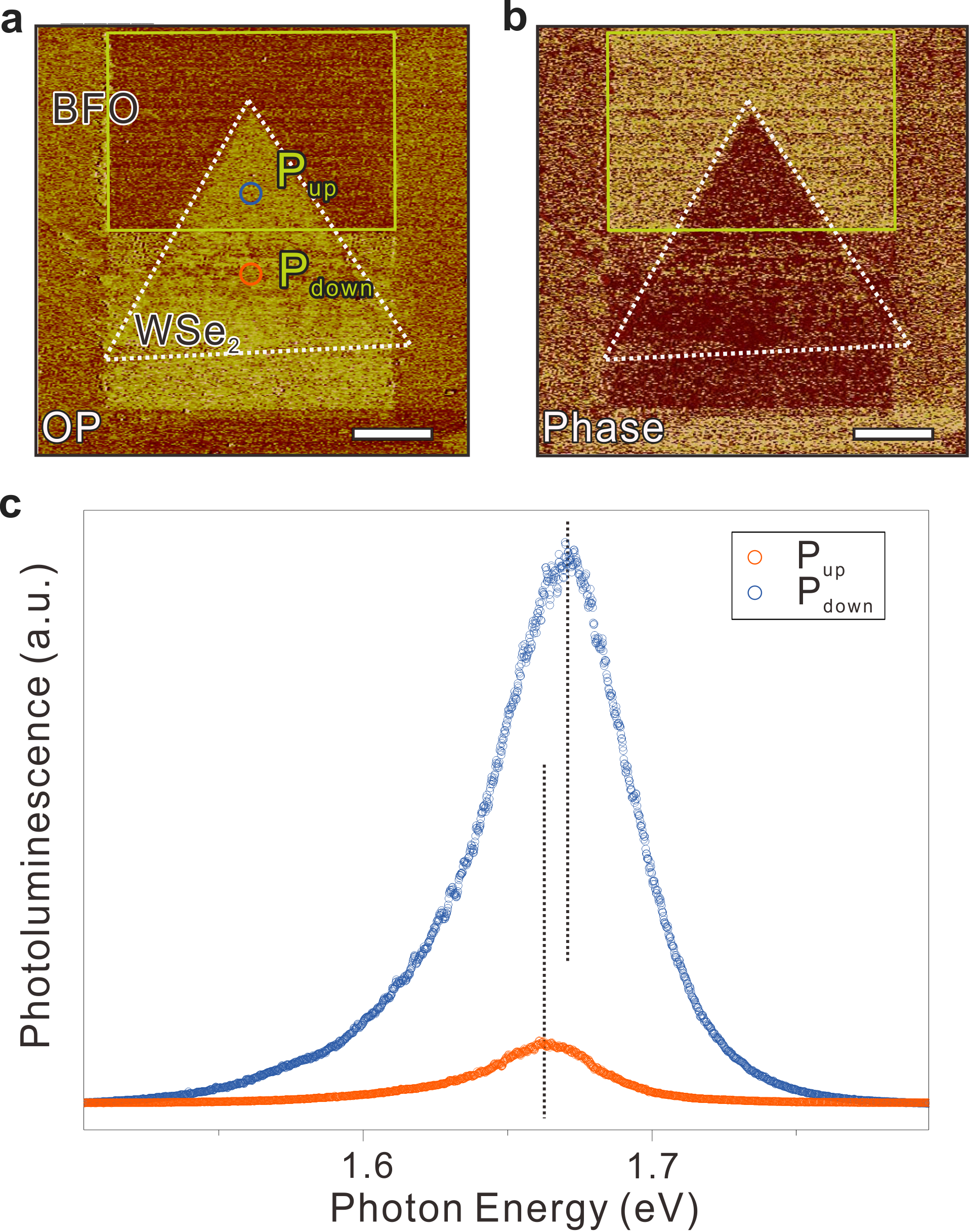
**

**Supplementary Figure 2**: (a)-(b) Piezoelectric-force microscope measured morphology, out-of-plane phase, and (c) photoluminescence from *P*_up_ and *P*_down_ regions of a WSe_2_/BFO system. Both scale bars in the figures are 5 μm.

**Supplementary Note 3: Band diagrams of a WSe_2_/BFO homojunction with polarization states upward (*P*_up_) and downward (*P*_down_)**

**Bare WSe_2_ and BFO**

To determine the valence-band offset ${\Delta E}_{v}$of the WSe_2_-BFO homojunctions, we require to know the respective core-level (CL) position relative to the valence-band maximum (*E*_VBM_), which was measured from the epitaxial layers on WSe_2_ and BFO, respectively. The value ${(E_{VBM}^{{WSe}_{2}}-E_{W 4f_{7/2}}^{{WSe}_{2}})}_{{WSe}_{2}}=31.8$**^[2]^** from the WSe_2_ layer and the obtained value $\left( E_{VBM}^{\mathrm{BFO}}-E_{Bi 4f}^{\mathrm{BFO}} \right)_{BFO}=157.8 \mathrm{eV}$ from BFO layer in Supplementary Figure 3.


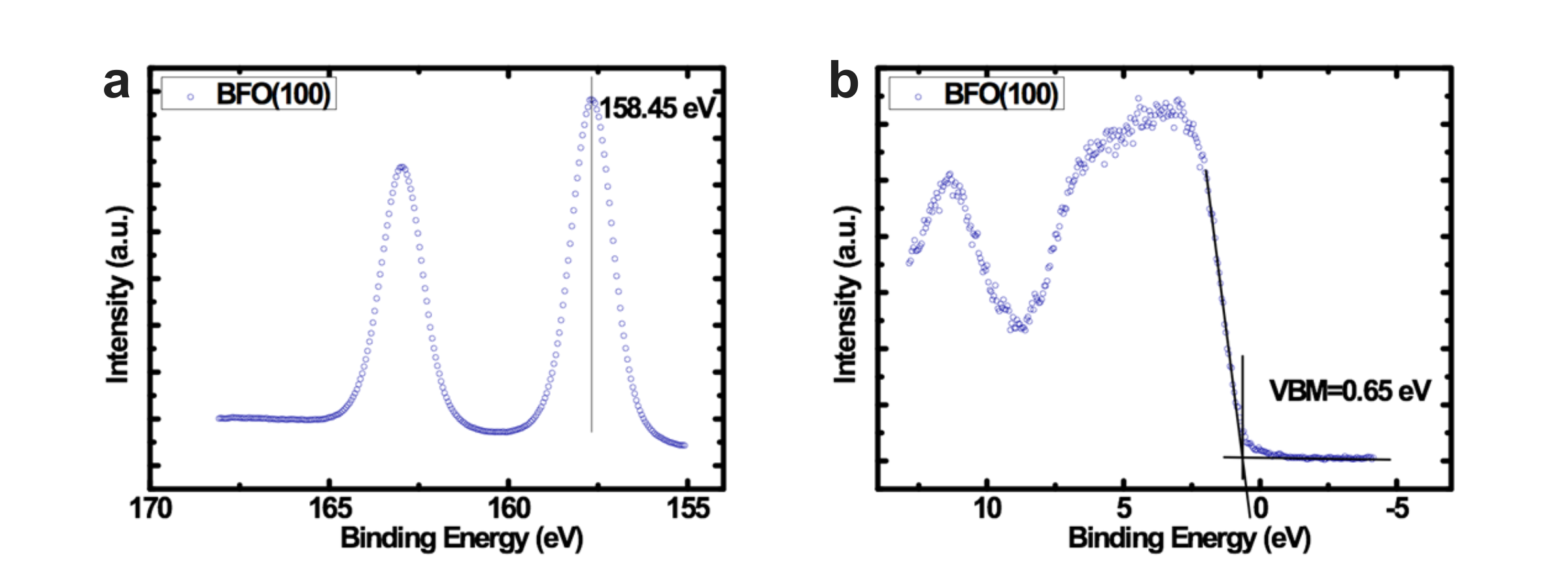


**Supplementary Figure 3**: (a)-(b) Bi 4*f* core-level and valence-band maximum in a bare BFO layer.

**WSe_2_ on BFO with polarization states *P*_up_ and *P*_down_**

As referred to in Fig. 3, the photoelectron emission spectra measured with SPEM of states *P*_up_ and *P*_down_ of a WSe_2_-BFO homojunction is shown in the following Supplementary Figure 4; the energy separations of the Bi 4*f* and W 4*f* core levels are summarized in Supplementary Table 1. The relation between the valence-band offset and the PES measurement is

$${\Delta E}_{v}={(E_{VBM}^{{WSe}_{2}}-E_{W 4f_{7/2}}^{{WSe}_{2}})}_{{WSe}_{2}}-\left( E_{VBM}^{\mathrm{BFO}}-E_{Bi 4f}^{\mathrm{BFO}} \right)_{BFO}+\left( \Delta E_{CL} \right)_{{WSe}_{2}-BFO} (1)$$

in which $\left( \Delta E_{CL} \right)_{{WSe}_{2}-BFO}$ is the energy separation between$\mathrm{Bi} 4f_{7/2}$ and $W 4f_{7/2},$and the two epitaxial layers are ${(E_{VBM}^{{WSe}_{2}}-E_{W 4f_{7/2}}^{{WSe}_{2}})}_{{WSe}_{2}}$ and $\left( E_{VBM}^{\mathrm{BFO}}-E_{Bi 4f}^{\mathrm{BFO}} \right)_{BFO}$

On combining the valence-band maximum (VBM) to the core-level value from bare WSe_2_ and BFO epilayers, we measured the apparent valence-band offset (VBO) values of *P*_up_ and *P*_down_ samples to be 0 and 1.0 eV, respectively. The band gap of WSe_2_ is known to be 1.6 eV**^[3,4]^**. We compared the value between W 4*f* core-level to valence-band maximum from Bare WSe_2_ layer (31.8 eV) and W 4*f* core-level positions from WSe_2_-BFO homojunction (32.2 eV for state *P*_down_, 33.2 eV for state *P*_up_), which reveals the Fermi level to be at 0.4 eV above the valence-band maximum for state *P*_down_ and 0.2 eV below the conduction-band minima for state *P*_up_. This *pn* junction formed for WSe_2_ placed on a BFO layer is shown in Supplementary Figures 4 (d) and (e).


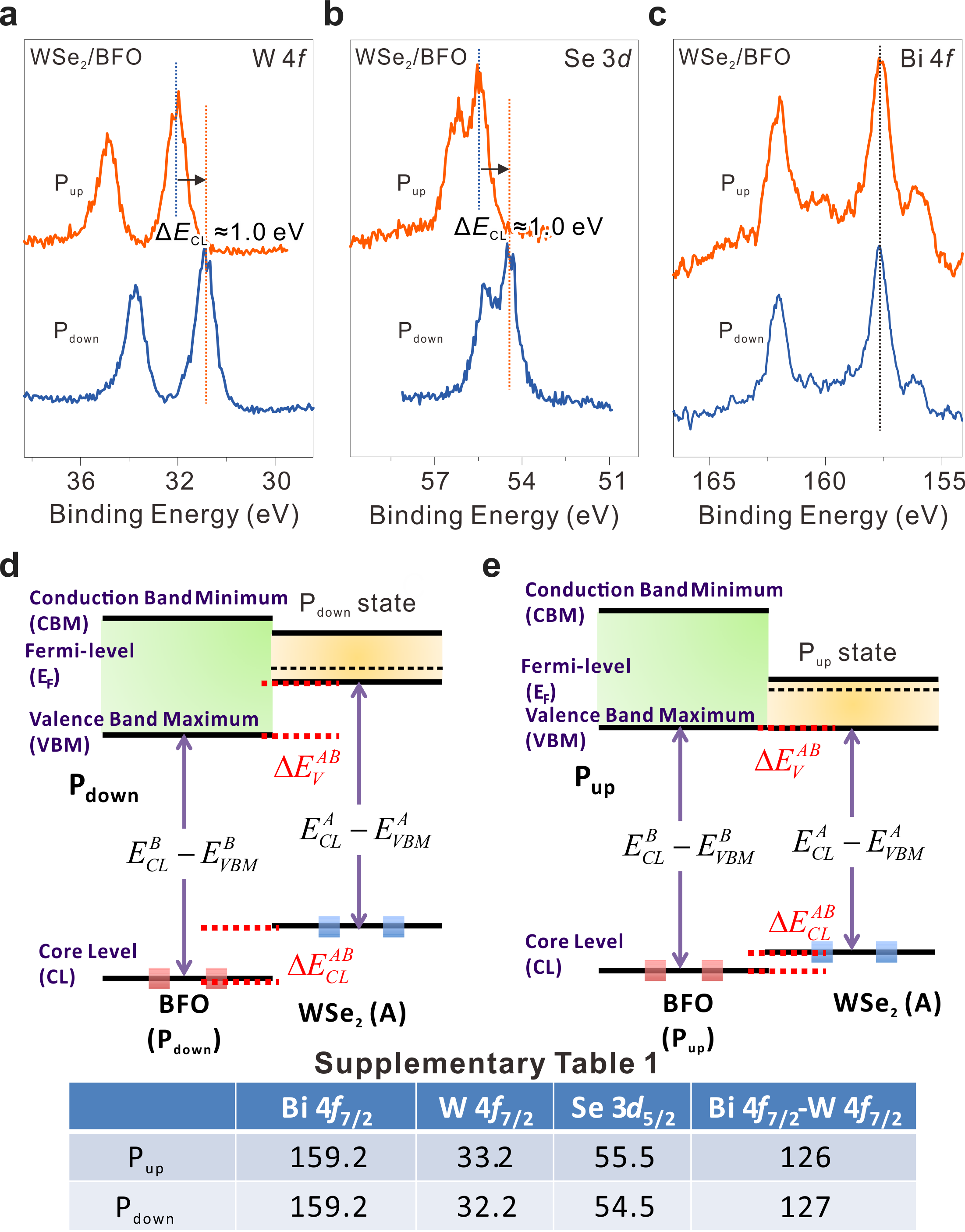


**Supplementary Figure 4**: (a)-(c) W 4*f*, Se 3*d* and Bi 4*f* core-level photoelectron spectra in regions *P*_up_ and *P*_down_ of a WSe_2_/BFO homojunction. (d)-(e) Band diagrams for regions *P*_up_ and *P*_down_ of a WSe_2_/BFO homojunction; *E_F_* denotes the Fermi level, *E_C_* is the conduction-band minimum, *E_V_* is the valence-band maximum, *E_CL_-E_VBM_* is the energy separation from the core-level to the valence-band maximum (VBM). **Supplementary Table 1** shows a summary of the Bi 4*f*_7/2_, W 4*f*_7/2_ and Se 3*d*_5/2_ core-level positions and energy difference between Bi 4*f* _7/2_ to W 4*f_7/2_* in various polarized WSe_2_/BFO samples.

**Supplementary Note 4: Effect of the substrate revealed in current-to-voltage and valence-band maxima for a WSe_2_/BFO homojunction**

Supplementary Figure 5 shows the substrate effect from a BFO layer in the transport electrical contact and XPS measurement. To reveal the *pn* junction with substrate effect, the sample was placed in a SEM chamber and transport measurement was performed with a conducting probe as shown in the inset of Supplementary Figure 5 (a). We measured a *pn* junction and BFO layer, but the current signal is obviously affected significantly by the BFO substrate in a region of negative bias. In addition, XPS measured valence band maxima pinning with respect to *P*_up_, *P*_down_ and bare BFO samples, respectively, which also revealed the substrate effect resulting from bare BFO layer.


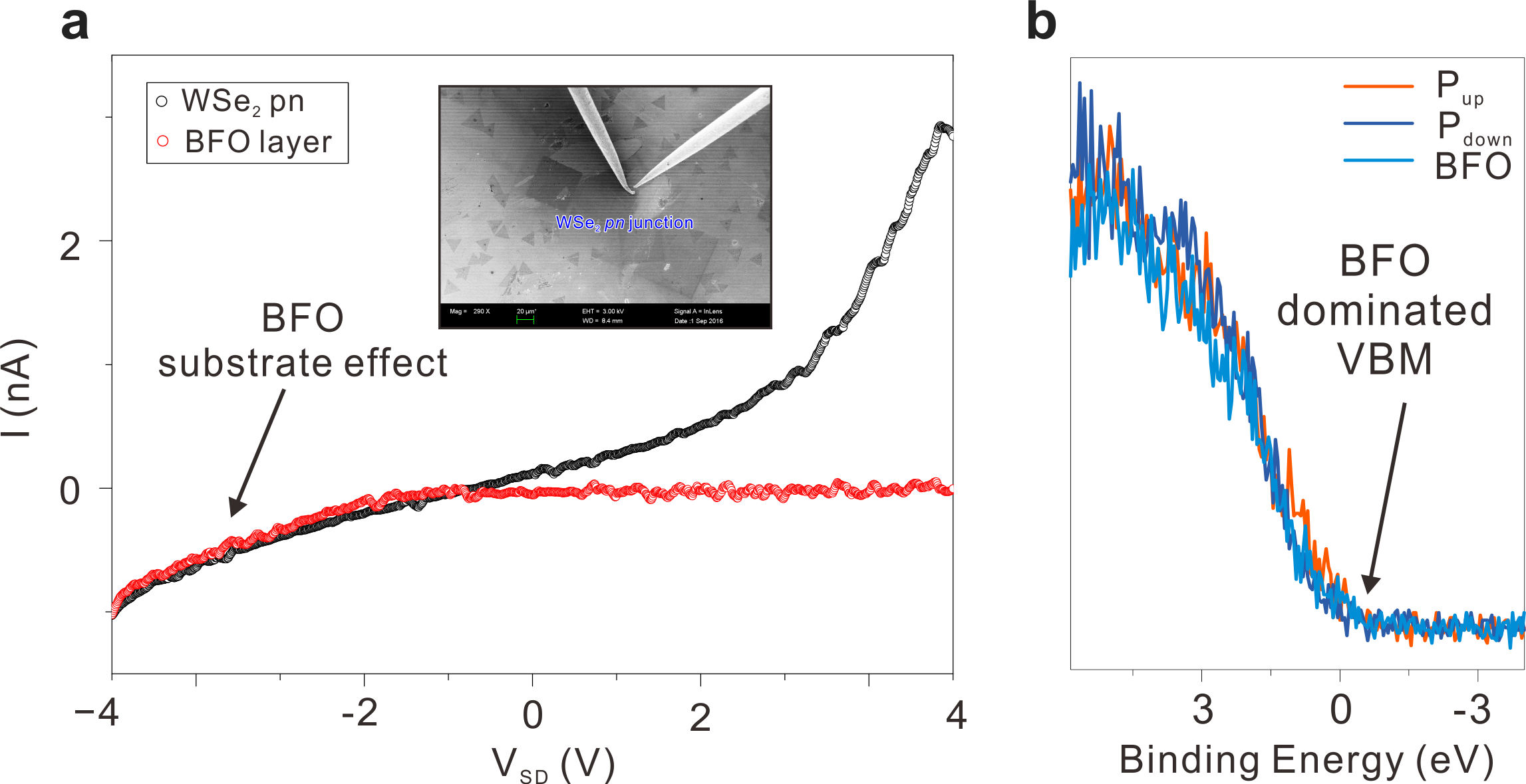


**Supplementary Figure 5**: (a) Current vs. voltage measured for the *pn* junction and a bare BFO layer. (b) XPS measurement of valence-band maxima pinning for *P*_up_, *P*_down_ and bare BFO layer.

**Supplementary Note 5: Transport behavior of *pp*, *nn* and *pn* junctions revealed in current-to-voltage for a WSe_2_/BFO homojunction (Diode-H)**

Figure 6 shows a current measurement for *pp*, *nn* and *pn* WSe_2_ junctions in the transport electrical contact measurement. We combine band structure in SPEM and electron affinity of WSe_2_**^[5]^**, and the resulting work function of Pd and WSe_2_ are values of about Φ_Pd_ = 5.2~5.6 eV, Φ*_p_*_-type_ = 5.3 ± 0.1 eV and Φ*_n_*_-type_ = 4.3 ± 0.1 eV. Thus, the *pp* and *nn* junctions show ohmic contact and Schottky contact behavior in Supplementary Figure 6, respectively. This transport result is not only consistent with our band structure prediction in SPEM, but also confirms the functionality of WSe_2_ homojunction.

**
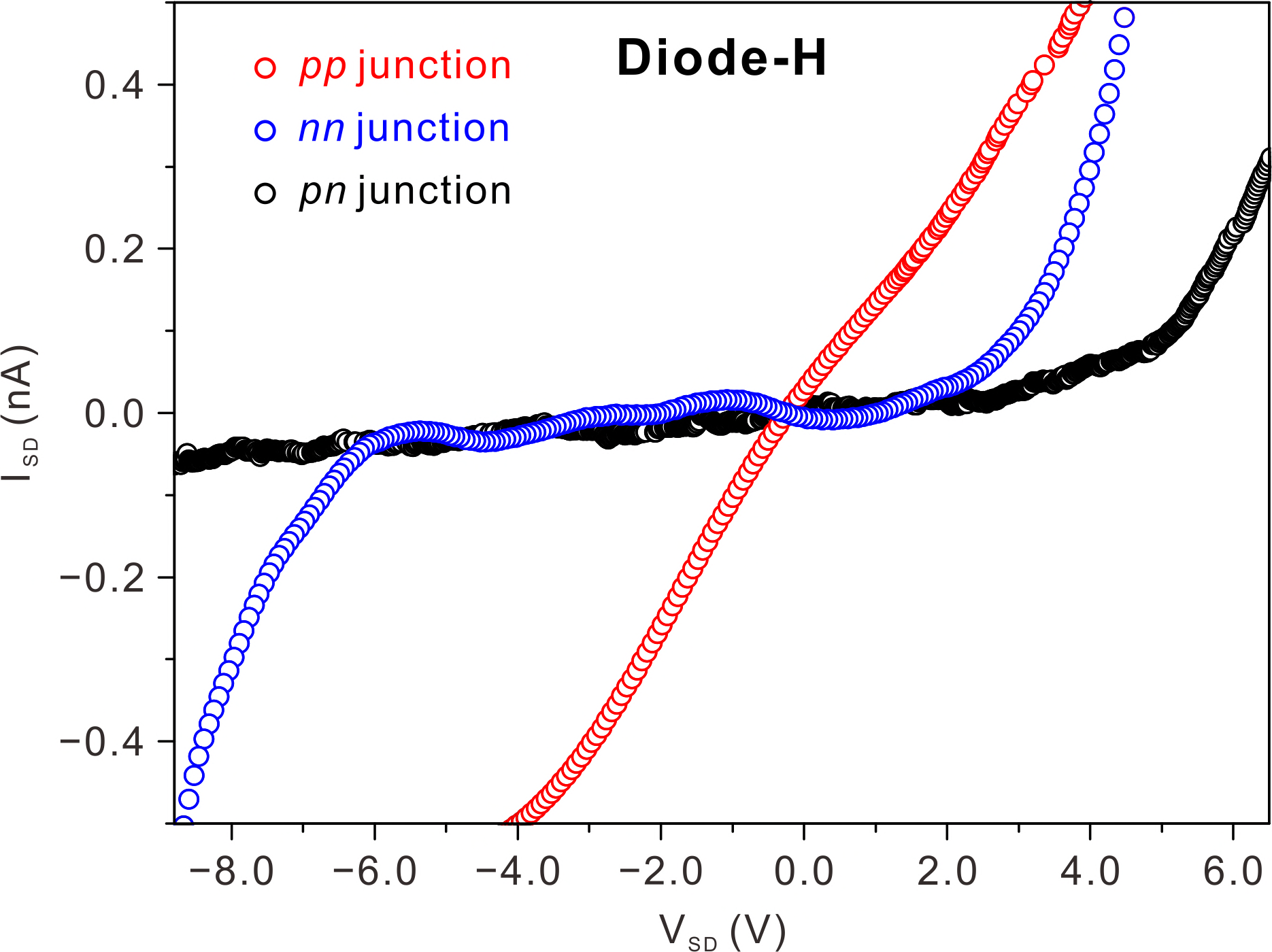
**

**Supplementary Figure 6**: Current vs. voltage measured in *pp*, *nn* and *pn* junctions in WSe_2_/BFO system.

**Supplementary Note 6: Carrier densities calculations**

**Estimation of carrier density from** **band alignment in SPEM**

1. We consider the electron density in conduction band and valence band**^[6]^**

*σ_n_ =(g_2D_k_B_T)ln{1+exp[(E_F_-E_C_)/ k_B_T]}* (2)

*σ_p_ =(g_2D_k_B_T)ln{1+exp[-(E_F_-E_V_)/ k_B_T]}* (3)

in which *g_2D_ =(4πm_e/h_^*^/h^2^)* is 2D density of states in TMD semiconductor (*m_e/h_** effective electron/hole mass, *E_F_-E_C_* and *E_F_-E_V_* are the separation from Fermi level to conduction band minima (CBM) and valence band maxima (VBM) measured with a SPEM, respectively

1. To estimate the electron/hole density of states, the effective mass of electron *m_e,WSe2_^*^=*0.33*m_0_* and hole *m_h,WSe2_^*^=*0.46 *m_0_* from WSe_2_ are used to estimate**^[7]^**

*g_2D_ =(4πm_e/h_^*^/h^2^)* (4)

=(4*πm_e/h_^*^*/(6.626×10^-34^)^2^)

1. Then we can get *n* of WSe_2_

*σ_n_ =(g_2D_k_B_T)ln{1+exp[(E_F_-E_C_)/ k_B_T]}*

*=(*4*π* ×*0.33*×9.11×10^-31^ ×1.38×10^-23^×300/(6.626×10^-34^)^2^*) ln{1**+exp[-0.2/*0.0257 *]}*

*= 3.56*×10^16^ cm^-2^× 4.17×10^-4^

~ *1.48*×10^13^ cm^-2^

*σ_p_ =(g_2D_k_B_T)ln{1+exp[-(E_F_-E_V_)/ k_B_T]}*

*=(*4*π* ×*0.46*×9.11×10^-31^ ×1.38×10^-23^×300/(6.626×10^-34^)^2^*) ln{1+exp[-0.4/*0.0257 *]}*

*= 4.96*×10^16^ cm^-2^× 1.74×10^-7^

~ 8.63×10^9^ cm^-2^

**Estimation of intrinsic carrier density from band alignment**

To illustrate how different types of carrier densities change in semiconductor, we have to introduce the intrinsic carrier density. By using mass action law in semiconductor, we can get the intrinsic carrier densities (*σ_i_*) as,

*σ_i_ =(g_2D,_**_h_k_B_T g_2D,e_k_B_T)^0.^**^5^* *exp[-E_g_/ 2k_B_T]*

*=(4.96*×10^16^×*3.56*×10^16^*)^0.5^ exp[-1.6/ 0.0514]*

*=4.20*×10^16^ cm^-2^×3×10^-14^

*=1.26*×10^3^ cm^-2^

The change of carrier density corresponds to the Fermi-level tuning in the band structure from SPEM, which shows 2D material can tune from intrinsic (*~*10^3^ cm^-2^) into *p*-type (~10^10^ cm^-2^) or *n*-type (*~*10^13^ cm^-2^) semiconductor. This reveals the carrier density manipulation is fully affected by BFO layer, which shows that a huge range of carrier density tuning is achievable.

**Supplementary References**

1. Y. –H. Chu, Q. Zhan, L. W. Martin, M. P. Cruz, P. –L. Yang, G. –W. Pabst, F. Zavaliche, S. –Y. Yang, J. –X. Zhang, L. –Q. Chen, D. G. Schlom, I. –N. Lin, T. –B. Wu and R. Ramesh, Advanced Materials 2006, **18**, 2307.
2. M. –H. Chiu, C. Zhang, H. –W. Shiu, C. –P. Chuu, C. –H. Chen, C. –Y. S. Chang, C. –H. Chen, M. –Y. Chou, C. –K. Shih and L. –J. Li, Nature Communications 2015, **6**, 7666.
3. B. Liu, Y. Ma, A. Zhang, A. N. Abbas, Y. Liu, C. Shen, H. Wan and C. Zhou, ACS Nano 2016, **10(5)**, 5153.
4. W. Liu, J. Kang, D. Sarkar, Y. Khatami, D. Jena and K. Banerjee, Nano Lett. 2013, **13**, 1983.
5. Christopher M. Smyth, R. Addou, S. McDonnell, Christopher L Hinkle, Robert M Wallace, 2D Materials 2017, **4**, 025084
6. N. Ma and D. Jena, 2D Materials 2015, **2**, 015003.
7. W. Liu, W. Cao, J. Kang and K. Banerjee, ECS Transactions 2013, **58(7)**, 281.
